# Bis{*N* ^2^,*N* ^6^-bis­[(pyridin-3-yl)meth­yl]pyridine-2,6-dicarboxamide-κ*N*}bis­(methanol-κ*O*)bis­(thio­cyanato-κ*N*)cobalt(II)

**DOI:** 10.1107/S1600536812020326

**Published:** 2012-05-12

**Authors:** Guang-Rui Yang, Juan Ren, Guo-Ting Li

**Affiliations:** a Department of Environmental and Municipal Engineering, North China University of Water Conservancy and Electric Power, Zhengzhou 450011, People’s Republic of China; b Henan Vocational College of Chemical Technology, Zhengzhou 450052, People’s Republic of China

## Abstract

In the title compound, [Co(NCS)_2_(C_19_H_17_N_5_O_2_)_2_(CH_3_OH)_2_], the Co^II^ atom lies on an inversion center and is coordinated by two isothio­cyanate N atoms, two O atoms of methanol mol­ecules and two pyridine N atoms in a slightly distorted octa­hedral environment. Inter­molecular O—H⋯O and N—H⋯N hydrogen bonds join the complex mol­ecules into layers parallel to the *bc* plane.

## Related literature

For the coordination chemistry of pyridyl­carboxamides, see: Thompson (2002 ▶); Wu *et al.* (2008 ▶). For the architectures of complexes with pyridyl­carboxamide ligands and various metal ions, see: Uemura *et al.* (2002 ▶); Burchell *et al.* (2006 ▶).
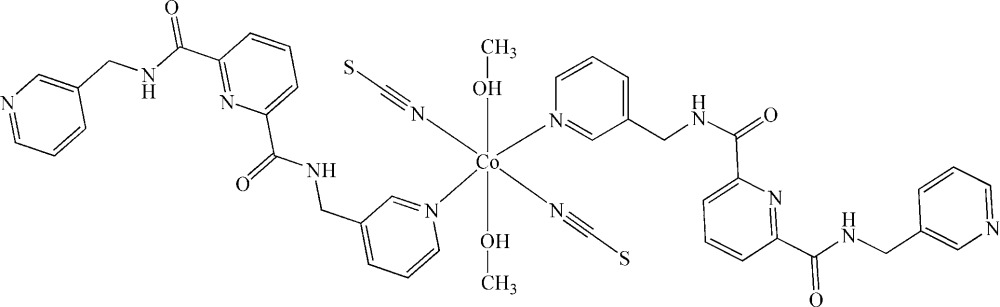


## Experimental

### 

#### Crystal data

[Co(NCS)_2_(C_19_H_17_N_5_O_2_)_2_(CH_4_O)_2_]
*M* *_r_* = 933.93
Monoclinic, 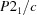

*a* = 9.6728 (19) Å
*b* = 17.631 (4) Å
*c* = 13.041 (3) Å
β = 100.13 (3)°
*V* = 2189.4 (8) Å^3^
*Z* = 2
Mo *K*α radiation
μ = 0.55 mm^−1^
*T* = 293 K
0.22 × 0.21 × 0.18 mm

#### Data collection

Siemens SMART CCD diffractometer
Absorption correction: multi-scan (*SADABS*; Sheldrick, 1996 ▶) *T*_min_ = 0.892, *T*_max_ = 0.914
21676 measured reflections
3803 independent reflections
3435 reflections with *I* > 2σ(*I*)
*R*_int_ = 0.049

#### Refinement

*R*[*F* ^2^ > 2σ(*F* ^2^)] = 0.054
*wR*(*F* ^2^) = 0.101
*S* = 1.15
3803 reflections
291 parameters
H atoms treated by a mixture of independent and constrained refinement
Δρ_max_ = 0.18 e Å^−3^
Δρ_min_ = −0.18 e Å^−3^

### 

Data collection: *SMART* (Siemens, 1996 ▶); cell refinement: *SAINT* (Siemens, 1996 ▶); data reduction: *SAINT*; program(s) used to solve structure: *SHELXS97* (Sheldrick, 2008 ▶); program(s) used to refine structure: *SHELXL97* (Sheldrick, 2008 ▶); molecular graphics: *SHELXTL* (Sheldrick, 2008 ▶); software used to prepare material for publication: *SHELXL97*.

## Supplementary Material

Crystal structure: contains datablock(s) I, global. DOI: 10.1107/S1600536812020326/yk2057sup1.cif

Structure factors: contains datablock(s) I. DOI: 10.1107/S1600536812020326/yk2057Isup2.hkl

Additional supplementary materials:  crystallographic information; 3D view; checkCIF report

## Figures and Tables

**Table 1:** Selected bond lengths (Å)

Co1—N6	2.074 (2)
Co1—O3	2.134 (2)
Co1—N4	2.162 (2)

**Table 2:** Hydrogen-bond geometry (Å, °)

*D*—H⋯*A*	*D*—H	H⋯*A*	*D*⋯*A*	*D*—H⋯*A*
N3—H3*A*⋯N5^i^	0.88	2.18	2.980 (3)	151
O3—H1⋯O2^ii^	0.76 (3)	1.94 (3)	2.679 (3)	163 (3)

Symmetry codes: (i) 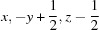; (ii) 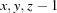.

**Fig. 1.**
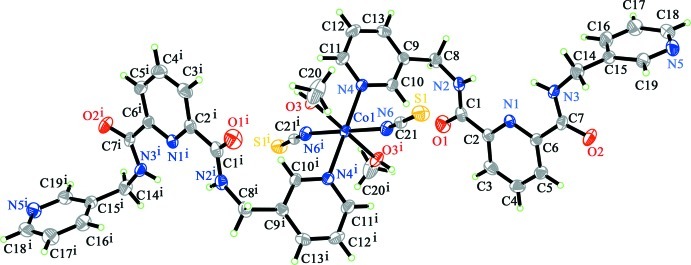
Diagram of the title compound with atom numbering scheme. Thermal ellipsoids are drawn at the 30% probability level. Symmetry code: (i) -x, -y, -z + 1.

**Fig. 2.**
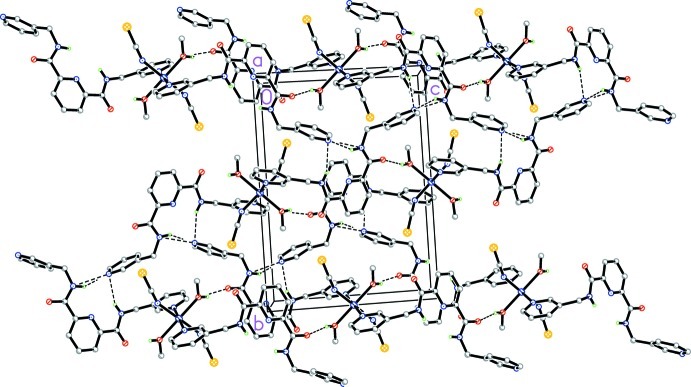
View of the two-dimensional network in the title compound formed by O—H···O and N—H···N hydrogen bonds.

## supplementary crystallographic information

### Comment

Pyridylcarboxamides derived from carboxylic acids form a class of spectacularly multidentate heterocyclic ligands and hold an important position in biochemistry and coordination chemistry (Thompson, 2002; Wu *et al.*, 2008). Over the last decades, several research groups worldwide have provided a wide range of structural motifs from isolated macrocycles, helicates to dynamic porous frameworks based on pyridylcarboxamide ligands (Uemura, *et al.*, 2002; Burchell, *et al.*, 2006). In course of such studies, we synthesized the symmetric multifunctional ligand *N*^2^,*N*^6^-bis((pyridin-3-yl)methyl)pyridine-2,6-dicarboxamide (BPDA) and prepared complexes of BPDA with some metal ions. Here we present the structure of such complex, [Co(BPDA)_2_(CH_4_O)_2_(SCN)_2_] (1).

The title compound is a mononuclear complex, where the Co^2+^ ion lies at the inversion center, thus the asymmetric unit consists of Co atom, one BPDA, one methanol molecule, and one SCN^-^ anion (Fig. 1). In (1) the coordination center is ligated by two isothiocyanato N atoms, two methanol O atoms, and two BPDA acting as monodentate ligands through their pyridyl N atoms. The octahedral coordination environment is slightly distorted, the largest deviation of coordination angles from idealized values are 1.59 (9) °.

Further aggregation of complex molecules is formed by the multiple hydrogen-bonding between the dicarboxamide groups of BPDA (as donors) and the uncoordinated pyridyl groups of other BPDA (as acceptors) as well as between the coordination methanol molecules (as donors) and the dicarboxamide groups of BPDA (as acceptors) (Table 2). Consequently, monomers are linked by O—H···O and N—H···N hydrogen bonds into a two-dimensional network parallel to the *bc* plane (Fig. 2). The layer structure is stabilized by face-to-face π···π stacking interactions between adjacent central pyridine rings of BPDA with a centroid to centroid distance of 3.793 (2) Å. Notably, that the ligand BPDA in (1) have pseudo-C_2_ symmetry and adopts helical conformation with the dihedral angles of the pendant pyridyl groups with the central pyridine ring of 76.1 (3) and 75.6 (3) °, respectively.

### Experimental

Synthesis of BPDA ligand. A mixture of 2,6-pyridinedicarboxylic acid (10 g, 60 mmol) and thionyl chloride (75 ml) was heated with reflux for 6 h under anhydrous condition, and then excess thionyl chloride was removed by rotary evaporation. The resulting white solid pyridine-2,6-dicarboyl dichloride was dissolved in dry CH_2_Cl_2_ (50 ml), to which a solution of 3-(aminomethyl)pyridine (13 g, 120 mmol) and triethylamine (24 ml) in dry CH_2_Cl_2_ (70 ml) was added dropwise with continuous stirring in an ice-bath. Stirred at room temperature for another hour, the mixture was washed with water (500 ml). The separated organic phase was dried with magnesium sulfate, and the solvent was removed by rotary evaporation. After recrystallization from alcohol/water (2:1), white crystals of BPDA were obtained (Yield: 70%). Selected IR (cm^-1^, KBr pellet): 3551(*m*), 3305(*s*), 3055(*m*), 2925(*m*), 1670(*vs*), 1593(*m*), 1542(*vs*), 1478(*m*), 1425(*m*), 1313(*m*), 1258(*m*), 1175(*m*), 1076(*m*), 1000(*s*), 864(*m*), 770(*s*), 679(*m*), 614(w).

The title compound (1) was prepared according to the following process. A solution of BPDA (69.4 mg, 0.2 mmol) in DMF (5 ml) was dropwise added into a solution of CoSO_4_.6H_2_O (28.1 mg, 0.1 mmol) in methanol (5 ml), and then a solution of KSCN (19.4 mg, 0.2 mmol) in methanol (5 ml) was dropwise added into the above mixture. With stirring for 30 minutes, the resulting mixture was filtered. The filtrate was allowed to evaporate at room temperature for two days, and pink crystals were obtain in 48% yield. Selected IR (cm^-1^, KBr pellet): 3351(*m*), 2072(*vs*), 1670(*vs*), 1534(*vs*), 1437(*m*), 1087(*m*), 750(*m*), 709(*m*).

### Refinement

Two very strong reflections, (2 1 1) and (-1 4 1), were omitted because of intensity overflow. All H atoms attached to the C and N atoms were positioned geometrically at distances 0.98 Å (CH_3_), 0.99 Å (CH_2_), 0.95 Å (CH) and 0.88 Å (NH) and refined using a riding model with *U_iso_*(H) = 1.2*U_eq_*(C,N) and *U_iso_*(H) = 1.5*U_eq_*(C_methyl_). The positional parameters of the H atom attached to oxygen were refined freely, and at the last stage of the refinement they were restrained with the H—O = 0.82 (3) Å and with *U_iso_*(H) = 1.2*U_eq_*(O).

### Crystal data

**Table:**

[Co(NCS)_2_(C_19_H_17_N_5_O_2_)_2_(CH_4_O)_2_]	*F*(000) = 970
*M**_r_* = 933.93	*D*_x_ = 1.417 Mg m^−^^3^
Monoclinic, *P*2_1_/*c*	Mo *K*α radiation, λ = 0.71073 Å
Hall symbol: -P 2ybc	Cell parameters from 4895 reflections
*a* = 9.6728 (19) Å	θ = 2.1–30.8°
*b* = 17.631 (4) Å	µ = 0.55 mm^−^^1^
*c* = 13.041 (3) Å	*T* = 293 K
β = 100.13 (3)°	Block, pink
*V* = 2189.4 (8) Å^3^	0.22 × 0.21 × 0.18 mm
*Z* = 2	

### Data collection

**Table:**

Siemens SMART CCD diffractometer	3803 independent reflections
Radiation source: fine-focus sealed tube	3435 reflections with *I* > 2σ(*I*)
Graphite monochromator	*R*_int_ = 0.049
ω scan	θ_max_ = 25.0°, θ_min_ = 2.4°
Absorption correction: multi-scan (*SADABS*; Sheldrick, 1996)	*h* = −11→11
*T*_min_ = 0.892, *T*_max_ = 0.914	*k* = −20→20
21676 measured reflections	*l* = −15→15

### Refinement

**Table:**

Refinement on *F*^2^	Primary atom site location: structure-invariant direct methods
Least-squares matrix: full	Secondary atom site location: difference Fourier map
*R*[*F*^2^ > 2σ(*F*^2^)] = 0.054	Hydrogen site location: inferred from neighbouring sites
*wR*(*F*^2^) = 0.101	H atoms treated by a mixture of independent and constrained refinement
*S* = 1.15	*w* = 1/[σ^2^(*F*_o_^2^) + (0.0359*P*)^2^ + 0.829*P*] where *P* = (*F*_o_^2^ + 2*F*_c_^2^)/3
3803 reflections	(Δ/σ)_max_ < 0.001
291 parameters	Δρ_max_ = 0.18 e Å^−^^3^
0 restraints	Δρ_min_ = −0.18 e Å^−^^3^

### Special details

**Table:**

Geometry. All s.u.'s (except the s.u. in the dihedral angle between two l.s. planes) are estimated using the full covariance matrix. The cell s.u.'s are taken into account individually in the estimation of s.u.'s in distances, angles and torsion angles; correlations between s.u.'s in cell parameters are only used when they are defined by crystal symmetry. An approximate (isotropic) treatment of cell s.u.'s is used for estimating s.u.'s involving l.s. planes.
Refinement. Refinement of *F*^2^ against ALL reflections. The weighted *R*-factor *wR* and goodness of fit *S* are based on *F*^2^, conventional *R*-factors *R* are based on *F*, with *F* set to zero for negative *F*^2^. The threshold expression of *F*^2^ > σ(*F*^2^) is used only for calculating *R*-factors(gt) *etc*. and is not relevant to the choice of reflections for refinement. *R*-factors based on *F*^2^ are statistically about twice as large as those based on *F*, and *R*- factors based on ALL data will be even larger.

### Fractional atomic coordinates and isotropic or equivalent isotropic displacement parameters (Å^2^)

**Table:**

	*x*	*y*	*z*	*U*_iso_*/*U*_eq_	
Co1	0.0000	0.0000	0.5000	0.03328 (16)	
S1	−0.19041 (9)	0.20444 (5)	0.66597 (7)	0.0607 (3)	
O1	0.3791 (3)	−0.10454 (13)	0.85021 (19)	0.0727 (7)	
O2	0.0978 (2)	0.10649 (12)	1.22010 (16)	0.0622 (6)	
O3	−0.0365 (2)	0.08401 (12)	0.38047 (17)	0.0472 (5)	
N1	0.2436 (2)	0.02348 (12)	1.01554 (16)	0.0373 (5)	
N2	0.3961 (2)	0.02276 (14)	0.86373 (18)	0.0463 (6)	
H2A	0.3683	0.0635	0.8935	0.056*	
N3	0.2057 (2)	0.16022 (13)	1.09943 (17)	0.0443 (6)	
H3A	0.2468	0.1532	1.0451	0.053*	
N4	0.2142 (2)	0.03865 (13)	0.54496 (17)	0.0397 (5)	
N5	0.3318 (3)	0.30281 (16)	1.4128 (2)	0.0604 (7)	
N6	−0.0655 (3)	0.07304 (14)	0.60659 (18)	0.0458 (6)	
C1	0.3501 (3)	−0.04486 (18)	0.8894 (2)	0.0471 (7)	
C2	0.2582 (3)	−0.04329 (16)	0.9707 (2)	0.0409 (7)	
C3	0.1951 (3)	−0.10950 (17)	0.9980 (2)	0.0533 (8)	
H3	0.2049	−0.1558	0.9628	0.064*	
C4	0.1183 (3)	−0.10634 (19)	1.0772 (3)	0.0583 (9)	
H4	0.0753	−0.1508	1.0983	0.070*	
C5	0.1043 (3)	−0.03805 (18)	1.1255 (2)	0.0497 (8)	
H5	0.0521	−0.0346	1.1806	0.060*	
C6	0.1676 (3)	0.02553 (15)	1.0922 (2)	0.0386 (7)	
C7	0.1543 (3)	0.10093 (17)	1.1426 (2)	0.0419 (7)	
C8	0.4895 (3)	0.0323 (2)	0.7894 (2)	0.0536 (8)	
H8A	0.5457	−0.0145	0.7886	0.064*	
H8B	0.5554	0.0742	0.8134	0.064*	
C9	0.4171 (3)	0.04924 (16)	0.6792 (2)	0.0405 (7)	
C10	0.2796 (3)	0.02987 (16)	0.6441 (2)	0.0420 (7)	
H10	0.2275	0.0090	0.6926	0.050*	
C11	0.2886 (3)	0.06854 (17)	0.4779 (2)	0.0509 (8)	
H11	0.2453	0.0745	0.4071	0.061*	
C12	0.4252 (4)	0.0909 (2)	0.5080 (3)	0.0633 (9)	
H12	0.4747	0.1132	0.4588	0.076*	
C13	0.4904 (3)	0.08126 (19)	0.6091 (3)	0.0566 (9)	
H13	0.5853	0.0965	0.6306	0.068*	
C14	0.1958 (4)	0.23665 (17)	1.1399 (2)	0.0537 (8)	
H14A	0.0988	0.2452	1.1519	0.064*	
H14B	0.2145	0.2736	1.0867	0.064*	
C15	0.2965 (3)	0.25142 (15)	1.2399 (2)	0.0419 (7)	
C16	0.4374 (4)	0.23449 (18)	1.2524 (3)	0.0594 (9)	
H16	0.4747	0.2111	1.1975	0.071*	
C17	0.5232 (4)	0.2516 (2)	1.3443 (3)	0.0665 (10)	
H17	0.6207	0.2403	1.3541	0.080*	
C18	0.4669 (4)	0.28538 (19)	1.4224 (3)	0.0620 (9)	
H18	0.5273	0.2968	1.4861	0.074*	
C19	0.2503 (3)	0.28563 (17)	1.3221 (2)	0.0522 (8)	
H19	0.1533	0.2980	1.3139	0.063*	
C20	−0.1081 (5)	0.1531 (2)	0.3779 (3)	0.0936 (15)	
H20A	−0.1899	0.1473	0.4122	0.140*	
H20B	−0.1394	0.1686	0.3053	0.140*	
H20C	−0.0453	0.1919	0.4143	0.140*	
C21	−0.1174 (3)	0.12750 (16)	0.6318 (2)	0.0387 (6)	
H1	0.005 (3)	0.0815 (17)	0.336 (2)	0.045 (10)*	

### Atomic displacement parameters (Å^2^)

**Table:**

	*U*^11^	*U*^22^	*U*^33^	*U*^12^	*U*^13^	*U*^23^
Co1	0.0331 (3)	0.0375 (3)	0.0294 (3)	0.0044 (2)	0.0059 (2)	0.0039 (2)
S1	0.0654 (6)	0.0427 (5)	0.0790 (6)	0.0000 (4)	0.0261 (5)	−0.0131 (4)
O1	0.0898 (19)	0.0547 (14)	0.0772 (17)	0.0137 (13)	0.0244 (14)	−0.0174 (13)
O2	0.0804 (17)	0.0688 (15)	0.0449 (13)	0.0054 (12)	0.0318 (12)	0.0063 (11)
O3	0.0517 (13)	0.0519 (13)	0.0415 (12)	0.0129 (10)	0.0179 (11)	0.0156 (10)
N1	0.0372 (13)	0.0400 (13)	0.0322 (12)	0.0034 (10)	−0.0006 (10)	−0.0006 (10)
N2	0.0466 (15)	0.0544 (16)	0.0378 (13)	0.0025 (12)	0.0067 (11)	−0.0090 (11)
N3	0.0574 (16)	0.0449 (14)	0.0341 (13)	−0.0024 (12)	0.0176 (12)	−0.0015 (11)
N4	0.0358 (13)	0.0479 (14)	0.0352 (13)	−0.0013 (11)	0.0063 (10)	0.0022 (11)
N5	0.0644 (19)	0.0654 (18)	0.0547 (17)	−0.0038 (15)	0.0195 (15)	−0.0203 (14)
N6	0.0497 (15)	0.0484 (15)	0.0415 (14)	0.0059 (12)	0.0143 (12)	−0.0012 (12)
C1	0.0476 (18)	0.0490 (19)	0.0406 (17)	0.0103 (15)	−0.0035 (14)	−0.0062 (15)
C2	0.0404 (16)	0.0441 (17)	0.0344 (15)	0.0043 (13)	−0.0043 (12)	0.0014 (13)
C3	0.056 (2)	0.0406 (18)	0.057 (2)	0.0008 (15)	−0.0057 (16)	−0.0021 (15)
C4	0.060 (2)	0.051 (2)	0.062 (2)	−0.0100 (16)	0.0035 (17)	0.0104 (17)
C5	0.0493 (18)	0.057 (2)	0.0419 (17)	−0.0049 (15)	0.0047 (14)	0.0100 (15)
C6	0.0362 (15)	0.0459 (17)	0.0321 (15)	0.0021 (13)	0.0016 (12)	0.0063 (12)
C7	0.0413 (16)	0.0553 (19)	0.0288 (15)	0.0063 (14)	0.0057 (13)	0.0040 (13)
C8	0.0359 (17)	0.077 (2)	0.0457 (18)	−0.0008 (16)	0.0012 (14)	−0.0087 (16)
C9	0.0336 (15)	0.0451 (16)	0.0421 (16)	0.0006 (13)	0.0049 (13)	−0.0066 (13)
C10	0.0400 (16)	0.0530 (18)	0.0337 (15)	−0.0002 (14)	0.0082 (13)	0.0005 (13)
C11	0.0484 (18)	0.062 (2)	0.0426 (18)	−0.0015 (16)	0.0094 (15)	0.0125 (15)
C12	0.057 (2)	0.080 (2)	0.058 (2)	−0.0173 (18)	0.0216 (17)	0.0120 (18)
C13	0.0409 (18)	0.068 (2)	0.061 (2)	−0.0175 (16)	0.0086 (16)	−0.0059 (17)
C14	0.070 (2)	0.0449 (18)	0.0483 (18)	0.0091 (16)	0.0159 (16)	−0.0003 (15)
C15	0.0495 (18)	0.0343 (15)	0.0444 (17)	0.0026 (13)	0.0153 (14)	−0.0027 (13)
C16	0.063 (2)	0.061 (2)	0.060 (2)	0.0124 (17)	0.0266 (18)	−0.0089 (17)
C17	0.053 (2)	0.073 (2)	0.075 (3)	0.0072 (18)	0.0156 (19)	−0.009 (2)
C18	0.065 (2)	0.057 (2)	0.063 (2)	−0.0105 (18)	0.0105 (18)	−0.0089 (18)
C19	0.0488 (18)	0.0533 (19)	0.059 (2)	0.0015 (15)	0.0220 (16)	−0.0111 (16)
C20	0.148 (4)	0.068 (2)	0.072 (3)	0.060 (3)	0.040 (3)	0.029 (2)
C21	0.0366 (16)	0.0423 (16)	0.0377 (16)	−0.0074 (13)	0.0080 (12)	−0.0005 (13)

### Geometric parameters (Å, º)

**Table:**

Co1—N6^i^	2.074 (2)	C4—H4	0.9500
Co1—N6	2.074 (2)	C5—C6	1.384 (4)
Co1—O3	2.134 (2)	C5—H5	0.9500
Co1—O3^i^	2.134 (2)	C6—C7	1.499 (4)
Co1—N4^i^	2.162 (2)	C8—C9	1.513 (4)
Co1—N4	2.162 (2)	C8—H8A	0.9900
S1—C21	1.627 (3)	C8—H8B	0.9900
O1—C1	1.224 (3)	C9—C10	1.371 (4)
O2—C7	1.234 (3)	C9—C13	1.373 (4)
O3—C20	1.399 (4)	C10—H10	0.9500
O3—H1	0.76 (3)	C11—C12	1.369 (4)
N1—C2	1.333 (3)	C11—H11	0.9500
N1—C6	1.341 (3)	C12—C13	1.369 (4)
N2—C1	1.336 (4)	C12—H12	0.9500
N2—C8	1.447 (4)	C13—H13	0.9500
N2—H2A	0.8800	C14—C15	1.507 (4)
N3—C7	1.325 (3)	C14—H14A	0.9900
N3—C14	1.456 (4)	C14—H14B	0.9900
N3—H3A	0.8800	C15—C19	1.372 (4)
N4—C11	1.334 (3)	C15—C16	1.377 (4)
N4—C10	1.344 (3)	C16—C17	1.366 (5)
N5—C18	1.327 (4)	C16—H16	0.9500
N5—C19	1.335 (4)	C17—C18	1.372 (5)
N6—C21	1.158 (3)	C17—H17	0.9500
C1—C2	1.498 (4)	C18—H18	0.9500
C2—C3	1.393 (4)	C19—H19	0.9500
C3—C4	1.375 (4)	C20—H20A	0.9800
C3—H3	0.9500	C20—H20B	0.9800
C4—C5	1.377 (4)	C20—H20C	0.9800
			
N6^i^—Co1—N6	180.00 (9)	N3—C7—C6	116.4 (2)
N6^i^—Co1—O3	88.41 (9)	N2—C8—C9	114.9 (2)
N6—Co1—O3	91.59 (9)	N2—C8—H8A	108.6
N6^i^—Co1—O3^i^	91.59 (9)	C9—C8—H8A	108.6
N6—Co1—O3^i^	88.41 (9)	N2—C8—H8B	108.6
O3—Co1—O3^i^	180.0	C9—C8—H8B	108.6
N6^i^—Co1—N4^i^	90.80 (9)	H8A—C8—H8B	107.5
N6—Co1—N4^i^	89.20 (9)	C10—C9—C13	117.6 (3)
O3—Co1—N4^i^	89.60 (9)	C10—C9—C8	121.8 (3)
O3^i^—Co1—N4^i^	90.40 (9)	C13—C9—C8	120.4 (3)
N6^i^—Co1—N4	89.20 (9)	N4—C10—C9	123.9 (3)
N6—Co1—N4	90.80 (9)	N4—C10—H10	118.1
O3—Co1—N4	90.40 (9)	C9—C10—H10	118.1
O3^i^—Co1—N4	89.60 (9)	N4—C11—C12	122.0 (3)
N4^i^—Co1—N4	180.0	N4—C11—H11	119.0
C20—O3—Co1	129.8 (2)	C12—C11—H11	119.0
C20—O3—H1	111 (2)	C13—C12—C11	119.9 (3)
Co1—O3—H1	118 (2)	C13—C12—H12	120.1
C2—N1—C6	117.7 (2)	C11—C12—H12	120.1
C1—N2—C8	123.2 (3)	C12—C13—C9	119.2 (3)
C1—N2—H2A	118.4	C12—C13—H13	120.4
C8—N2—H2A	118.4	C9—C13—H13	120.4
C7—N3—C14	121.5 (2)	N3—C14—C15	113.6 (2)
C7—N3—H3A	119.2	N3—C14—H14A	108.9
C14—N3—H3A	119.2	C15—C14—H14A	108.9
C11—N4—C10	117.3 (2)	N3—C14—H14B	108.9
C11—N4—Co1	123.2 (2)	C15—C14—H14B	108.9
C10—N4—Co1	119.40 (18)	H14A—C14—H14B	107.7
C18—N5—C19	116.7 (3)	C19—C15—C16	117.1 (3)
C21—N6—Co1	154.7 (2)	C19—C15—C14	120.2 (3)
O1—C1—N2	123.5 (3)	C16—C15—C14	122.7 (3)
O1—C1—C2	121.3 (3)	C17—C16—C15	119.4 (3)
N2—C1—C2	115.2 (3)	C17—C16—H16	120.3
N1—C2—C3	122.9 (3)	C15—C16—H16	120.3
N1—C2—C1	116.7 (3)	C16—C17—C18	119.2 (3)
C3—C2—C1	120.5 (3)	C16—C17—H17	120.4
C4—C3—C2	118.5 (3)	C18—C17—H17	120.4
C4—C3—H3	120.7	N5—C18—C17	122.9 (3)
C2—C3—H3	120.7	N5—C18—H18	118.5
C3—C4—C5	119.3 (3)	C17—C18—H18	118.5
C3—C4—H4	120.4	N5—C19—C15	124.6 (3)
C5—C4—H4	120.4	N5—C19—H19	117.7
C4—C5—C6	118.7 (3)	C15—C19—H19	117.7
C4—C5—H5	120.7	O3—C20—H20A	109.5
C6—C5—H5	120.7	O3—C20—H20B	109.5
N1—C6—C5	122.9 (3)	H20A—C20—H20B	109.5
N1—C6—C7	116.9 (2)	O3—C20—H20C	109.5
C5—C6—C7	120.2 (3)	H20A—C20—H20C	109.5
O2—C7—N3	122.6 (3)	H20B—C20—H20C	109.5
O2—C7—C6	121.0 (3)	N6—C21—S1	179.4 (3)
			
N6^i^—Co1—O3—C20	167.7 (3)	C4—C5—C6—C7	179.8 (3)
N6—Co1—O3—C20	−12.3 (3)	C14—N3—C7—O2	−1.4 (4)
N4^i^—Co1—O3—C20	76.9 (3)	C14—N3—C7—C6	178.5 (2)
N4—Co1—O3—C20	−103.1 (3)	N1—C6—C7—O2	−173.0 (3)
N6^i^—Co1—N4—C11	57.5 (2)	C5—C6—C7—O2	6.5 (4)
N6—Co1—N4—C11	−122.5 (2)	N1—C6—C7—N3	7.2 (4)
O3—Co1—N4—C11	−30.9 (2)	C5—C6—C7—N3	−173.4 (3)
O3^i^—Co1—N4—C11	149.1 (2)	C1—N2—C8—C9	94.9 (3)
N6^i^—Co1—N4—C10	−119.7 (2)	N2—C8—C9—C10	−22.9 (4)
N6—Co1—N4—C10	60.3 (2)	N2—C8—C9—C13	160.6 (3)
O3—Co1—N4—C10	151.9 (2)	C11—N4—C10—C9	−0.5 (4)
O3^i^—Co1—N4—C10	−28.1 (2)	Co1—N4—C10—C9	176.9 (2)
O3—Co1—N6—C21	12.0 (5)	C13—C9—C10—N4	1.7 (4)
O3^i^—Co1—N6—C21	−168.0 (5)	C8—C9—C10—N4	−174.9 (3)
N4^i^—Co1—N6—C21	−77.6 (5)	C10—N4—C11—C12	−1.2 (4)
N4—Co1—N6—C21	102.4 (5)	Co1—N4—C11—C12	−178.4 (2)
C8—N2—C1—O1	−2.3 (4)	N4—C11—C12—C13	1.5 (5)
C8—N2—C1—C2	177.9 (2)	C11—C12—C13—C9	−0.2 (5)
C6—N1—C2—C3	1.8 (4)	C10—C9—C13—C12	−1.4 (5)
C6—N1—C2—C1	−176.7 (2)	C8—C9—C13—C12	175.3 (3)
O1—C1—C2—N1	173.9 (3)	C7—N3—C14—C15	74.6 (4)
N2—C1—C2—N1	−6.3 (4)	N3—C14—C15—C19	−133.0 (3)
O1—C1—C2—C3	−4.7 (4)	N3—C14—C15—C16	49.6 (4)
N2—C1—C2—C3	175.0 (3)	C19—C15—C16—C17	0.5 (5)
N1—C2—C3—C4	−2.2 (4)	C14—C15—C16—C17	178.0 (3)
C1—C2—C3—C4	176.3 (3)	C15—C16—C17—C18	0.0 (5)
C2—C3—C4—C5	1.0 (5)	C19—N5—C18—C17	0.2 (5)
C3—C4—C5—C6	0.4 (5)	C16—C17—C18—N5	−0.3 (5)
C2—N1—C6—C5	−0.3 (4)	C18—N5—C19—C15	0.3 (5)
C2—N1—C6—C7	179.1 (2)	C16—C15—C19—N5	−0.6 (5)
C4—C5—C6—N1	−0.8 (4)	C14—C15—C19—N5	−178.2 (3)

Symmetry code: (i) −*x*, −*y*, −*z*+1.

### Hydrogen-bond geometry (Å, º)

**Table:**

*D*—H···*A*	*D*—H	H···*A*	*D*···*A*	*D*—H···*A*
N3—H3*A*···N5^ii^	0.88	2.18	2.980 (3)	151
O3—H1···O2^iii^	0.76 (3)	1.94 (3)	2.679 (3)	163 (3)

Symmetry codes: (ii) *x*, −*y*+1/2, *z*−1/2; (iii) *x*, *y*, *z*−1.
